# Thirty-Seventh Annual Convention, Detroit, September, 1900

**Published:** 1900-12

**Authors:** 


### A. V. M. A.

### THIRTY-SEVENTH ANNUAL CONVENTION, DETROIT, SEPTEMBER, 1900.

(Continued from page 641.)

The Army Legislation Committee report was supplemented by an extended reference by Dr. R. S. Huidekoper to the work done and the direct opposition it had met with in the halls of Congress. The unjust and unfair criticisms of our profession were referred to, but he believed that they were largely due to utter ignorance of the advancement and growth of the profession in the last ten years, and that the special work this year of an educational character carried on all over the land through the profession would prove of very great value in the coming session of Congress. He stated his purpose of again reaching the officials of every State and local organization, with advice and information along the lines that would insure success at the next session. Dr. Hoskins followed with remarks very highly commending the leadership of Senator Kenney, of Delaware, on the floor of the Senate, and offering a hearty vote of thanks in recognition of his valued services, which was seconded and carried. Dr. Austin Peters followed in commendatory remarks of the earnest advocacy of our legislation by Senator Wolcott, of Colorado, to whom a very sincere vote of appreciation was extended. After some further remarks the appropriation of $250 to the Army Legislation Committee was adopted on recommendation of the Executive Committee.

The treasurer's report exhibited a very healthy condition of the finances of the company, showing receipts aggregating $1923.16 and expenditures of $1152.88, leaving a balance in bank of $771.92. One of the gratifying features of the treasurer's report was the large sums of money used by the Resident State Secretaries in their work of better organizing the profession in the several States for more efficient work in behalf of the profession. An increased balance of $470.10 over the preceding year, with very much larger expenses in certain directions, was reported.

Secretary Stewart, in a brief report, covered the work of that office for the year. The adoption of uniform stationery for all the officers and Resident State Secretaries had added much to the welfare of the Association. The gross collections for dues showed an increase of about $200 over the preceding year. He closed his report with the hope and a request that he be relieved from further services in the arduous post he had filled. Dr. Hoskins, in referring to the report and very efficient services rendered by Secretary Stewart, asked that a proposition upon the President's desk be taken up for consideration. The proposition recommended an increase of the Secretary's salary to three hundred dollars. It was signed by Drs. Reynolds, Winchester, Salmon, Clement, Pearson, Huidekoper, and Hoskins. On motion it was referred to the Executive Committee, and by them favorably approved and adopted at a subsequent session of the convention.

Dr. A. H. Baker, Chairman of the Committee on Intelligence and Education, made a short report on behalf of this committee, addressing his remarks specially to the work of the colleges. He caustically criticised the establishing of practitioners' courses by one of the leading colleges as dangerous and destined to work much injury in the progress of the profession. The report was followed by a very interesting discussion, participated in by Dr. Hoskins, President Pearson, Secretary Stewart, Dr. Williams, and Chairman Baker. The two-year schools and those establishing questionable practitioners' courses came in for a round of criticism.

The Librarian, Dr. W. L. Williams, gave a systematic report of the volumes of the proceedings in his keeping, the number distributed during the year, and of those to whom sent, including new members, health boards, publication committee, and others. He recommended for consideration a plan by which graduates and non-graduates ineligible for membership might obtain copies by an annual contribution.

(To be continued.)

